# Clinical pathway for patients with spondyloarthritis/Vía clínica para pacientes con espondiloartritis

**Published:** 2012-05-29

**Authors:** Loreto Carmona

**Affiliations:** Camilo José Cela University, Madrid, Spain

**Keywords:** delivery of health care, spondylitis, ankylosing, quality indicators, health care, referral and consultation, provisión de asistencia sanitaria, espondiloartritis, indicadores de calidad, asistencia sanitaria, derivación y consulta

## Introduction

Spondyloarthritis (SpA) affects approximately 1.9% of the general population and represents 13% of the consultations in rheumatology [1]. It particularly affects active people who are around 40 years of age, and in many cases leads to functional disability. In general, it is poorly understood and there is little awareness of the condition, leading to an unacceptable average delay in diagnosis of more than six years from the onset of symptoms, though this is subject to great inequalities and variability. Over the last decade, there have been many advances in this field, with new effective treatments, response and diagnostic criteria, and measurements and, accordingly, it is difficult for non-specialists to provide care according to the best scientific evidence.

Given all this, the Spanish Society of Rheumatology (SER) set three key objectives: 1) to improve the prognosis (functional status and quality of life of patients with SpA; 2) to facilitate early diagnosis; and 3) to promote research into recent-onset SpA. To this end, a care and research programme, called *Esperanza* (meaning “hope”, in Spanish), was established.

## Theory and methods

The general design of the programme arose from analysis of the status quo. The data required to determine the scope of the programme were obtained from the ESPIDEP study [2], a pilot programme of the Early-Onset SpA Unit, focused on training, referral and assessment, carried out in the catchment area of La Paz Hospital in Madrid over a six-month period.

The objectives of the ESPIDEP programme were to estimate the incidence of early-onset SpA and assess the usefulness of the system and criteria for referral. Referred patients had to be under 45 years of age, have inflammatory back pain or asymmetric arthritis of the lower limbs, and have a time since onset of symptoms of 3 to 24 months. The annual incidence was estimated to be 62.5 cases per 100,000 people and the system proved to be effective, there being an agreement between the criteria of the GPs and the rheumatologists in 82.7% of cases. Further, two-thirds of the patients referred had a final diagnosis of SpA.

The data necessary to validate the procedures were based on the SERAP programme of the SER, which was run in 34 health regions, for the detection of arthritis early after onset (referral + diagnosis + treatment) [3]. The programme included instructions and tools for referral, a diagnostic algorithm and flexible, evidence-based treatment recommendations. In SERAP, it was observed that the factors associated with greater efficiency of the units were feedback to primary care (PC), even negative feedback, and short waiting times for general rheumatology were short [4].

For designing the part of the programme that involves PC, data were obtained from a qualitative study involving GPs. This study investigated the beliefs and attitudes of GPs in relation to the application of a clinical pathway for recent-onset SpA in five focus groups [5]. As a result, the groundwork was laid for optimising collaboration with PC. The key elements identified were: simple and practical processes through official channels; as much feedback as possible, allowing rapid consultations and easy access to reports and guidance on treatment, particularly as regards to sick leave; and continuous medical training, preferably certified.

The programme has two components: management and research. The management component involves the establishment of standards, procedures (including the processes of referral and of specialised care, and continuous medical training) and continuous evaluation using quality indicators. The R & D plan establishes calls for proposals for research projects and backs specific projects. Both components are supported by a multidirectional electronic platform that monitors activity in real time.

## Results

A total of 25 units in 12 autonomous regions across Spain participated in the study, involving 1844 GPs and 1098 patients. The time elapsing between the visit to PC and the rheumatology appointment decreased from 40±25 to 11±8 days. Table 1 lists the quality indicators with their standards and outcomes to 15th November 2009. The calculations are not described here due to lack of space but are reported in detail in another recent publication concerning the programme [6].

## Discussion

*Esperanza* is the first health care programme on spondyloarthritis with a model for continuous assessment and improvement. By setting it up, we have been able to demonstrate that a programme based on specialised health care in the form of a clinical pathway is viable, and also that it is possible to introduce, assess and monitor the activity of such a programme, using scientific and management indicators. A large number of professionals and patients have benefit from this programme which has exceeded by far the initial objectives set. The standards must be regularly assessed to adapt to current conditions, but we believe that the *Esperanza* programme, with adjustments as necessary, is a model that could be extrapolated to other hospitals and diseases.

## Conference abstract Spanish

## Introducción

Las espondiloartritis (EspA) afectan aproximadamente al 1,9% de la población general y suponen un 13% de las consultas a reumatología [1]. Afectan sobre todo a población activa, con una edad media de 40 años y llevan a la incapacidad funcional a muchos de los pacientes. En general, se conocen poco y se está poco pendiente de ellas, lo que lleva a un retraso diagnóstico inaceptable, de más de 6 años tras el inicio de los síntomas, y sujeto a múltiples inequidades, con una gran variabilidad. En la última década se han dado muchos avances en este campo, con nuevos tratamientos eficaces, nuevos criterios de respuesta, mediciones, y criterios diagnósticos, hasta el punto que los no especialistas quedan muy limitados para ofrecer una atención en base a la mejor evidencia científica.

En base a estas premisas, la Sociedad Española de Reumatología (SER) se planteó tres objetivos clave: 1) mejorar el pronóstico (situación funcional y calidad de vida) de los pacientes con EspA, 2) facilitar el diagnóstico precoz y 3) estimular la investigación en EspA de inicio. Para ello estableció un programa asistencial y de investigación, denominado Esperanza.

## Teoría y métodos

El diseño general del programa partió de un análisis de la situación. Los datos necesarios para dimensionar el programa se obtuvieron del estudio ESPIDEP [2], un programa piloto de unidad de EspA precoz (UEsP) basado en formación, derivación y evaluación, llevado a cabo en el área de salud del Hospital La Paz de Madrid durante seis meses. Los objetivos de ESPIDEP eran estimar incidencia de EspA precoz y probar la utilidad de los criterios de derivación y el sistema. Los pacientes referidos debían tener menos de 45 años, dolor de espalda inflamatorio o artritis asimétrica de miembros inferiores y de 3 a 24 meses de duración de los síntomas. La incidencia anual estimada fue de 62,5 casos por 100.000 y el sistema se probó eficaz, existiendo una concordancia entre los criterios del Médico de Atención Primaria (MAP) y del reumatólogo del 82,7%. Dos tercios de los pacientes remitidos tenían un diagnóstico final de EspA.

Los datos necesarios para la validez de los procedimientos se basaron en el programa SERAP de la SER, realizado en 34 áreas de salud con el objetivo de detectar precozmente la artritis de inicio y establecer unidades de artritis de inicio (derivación+diagnóstico+tratamiento) [3]. El programa incluía instrucciones y materiales de derivación, un algoritmo diagnóstico y recomendaciones de tratamiento basadas en la evidencia y se caracterizaba por una gran flexibilidad. En SERAP se observó que los factores asociados a la mayor eficiencia de las unidades eran la retroalimentación a primaria, incluida la negativa y que los tiempos de espera a reumatología general fueran cortos [4].

Los datos necesarios para diseñar la vertiente del programa que afecta a Atención Primaria (AP), se obtuvieron de un estudio cualitativo a MAP. Este estudio exploró las creencias y actitudes de los MAP en la aplicación de una vía clínica a EspA de inicio en 5 grupos focales [5]. Como resultado, se obtuvieron las bases para una colaboración óptima con AP. Estas son: procesos sencillos y prácticos por vía oficial, con retroalimentación máxima que permitan consultas rápidas y acceso a informes y a recomendaciones sobre el tratamiento de cara a baja laboral y formación médica continuada, preferentemente acreditada.

El programa consta de dos planes, el gestor y el de investigación. El Plan gestor define el establecimiento de estándares, procedimientos (incluido el proceso de derivación y de cuidado especializado y la formación médica continua) y la evaluación continua mediante indicadores de calidad. El Plan de I+D, establece las convocatorias de proyectos de investigación y sigue proyectos específicos. Ambos se sustentan en una plataforma electrónica multidireccional que mide la actividad en tiempo real.

## Resultados

Se incluyeron 25 unidades en 12 comunidades autónomas, con 1844 MAP y 1098 pacientes. El tiempo entre la visita a AP y la de reumatología bajó de 40±25 a 11±8 días. La tabla 1 los indicadores de calidad con sus estándares y los resultados a 15 de noviembre de 2009. Los cálculos realizados se han suprimido por falta de espacio, pero pueden consultarse en la publicación más reciente del programa [6].

## Discusión

Esperanza es el primer programa asistencial en espondiloartritis que sigue el modelo de evaluación continua y mejora. Con su puesta en marcha, hemos podido demostrar que un programa de atención especializada con formato de vía clínica es viable, pero que además es posible implantar, evaluar y seguir la actividad de un programa como este a través de indicadores de gestión y científicos. Un gran número de profesionales y pacientes se han beneficiado de este programa que ha alcanzado con creces los objetivos propuestos a su inicio. Los estándares deben ser reevaluados periódicamente para adaptarse a la realidad, pero pensamos que el Programa Esperanza, realizando los ajustes necesarios, es un modelo exportable a más hospitales y patologías.

## Figures and Tables

**Table 1.** Quality indicators: standards and outcomes

	Name of the indicator	Objective	Standard	Value on 15 November 2009
1	Accessibility	See referred patients to the rheumatology unit with suspected SpA within 30 days	>90%	91%
2	Duration of the first visit	Provide adequate health care to patients with early-onset SpA	>50%	53%
3	Appropriateness of the referrals	Refer patients as appropriate and in line with the established protocol	>50%	92%
4	Success of referral criteria	Assess the usefulness of the criteria applied	>50%	28%
5	Standardised information	Provide patients with sufficient information to participate in the making of decisions related to their condition, and a diagnosis after being seen by specialists	>90%	25%
6	Communication of reports	Use of reports as feedback to GPs	>85%	2%
7	Adequate assessment	Perform basic examinations and tests to ensure the appropriate assessment of patients	<10%	24%
8	Regularity of check-ups	Ensure adequate monitoring	>90%	84%

**Tabla 1.** Indicadores de calidad: estándares y resultados

	Nombre del indicador	Objetivo	Estándar	Valor a 15/11/09
1	Accesibilidad	Recibir en la consulta de reumatología a los pacientes derivados con sospecha de EspA antes de que transcurran 30 días	>90%	91%
2	Duración de la primera visita	Proveer cuidado apropiado a pacientes con EspA precoz	>50%	53%
3	Adecuación de las derivaciones	Derivar a los pacientes a especializada de forma correcta en el formato preestablecido	>50%	92%
4	Éxito de los criterios de derivación	Evaluar la utilidad de los criterios utilizados	>50%	28%
5	Información normalizada	Que los pacientes dispongan de información suficiente para cooperar en la toma decisiones respecto a su enfermedad, y un diagnóstico tras ser atendidos en atención especializada	>90%	25%
6	Comunicación de informes	Utilización de los informes como retroalimentación al MAP	>85%	2%
7	Evaluación correcta	Mínimas exploraciones y medidas a realizar para asegurar una evaluación apropiada de los pacientes	<10%	24%
8	Periodicidad de revisión	Garantizar seguimiento adecuado	>90%	84%
